# Combination Therapy with GABA and MgSO_4_ Improves Insulin Sensitivity in Type 2 Diabetic Rat

**DOI:** 10.1155/2022/2144615

**Published:** 2022-02-15

**Authors:** Shahla Sohrabipour, Mohammad Reza Sharifi, Mohammadreza Sharifi, Ardeshir Talebi, Nepton Soltani

**Affiliations:** ^1^Endocrinology and Metabolism Research Center, Hormozgan University of Medical Sciences, Bandar Abbas, Iran; ^2^Department of Physiology, School of Medicine, Isfahan University of Medical Sciences, Isfahan, Iran; ^3^Department of Genetics and Molecular Biology, School of Medicine, Isfahan University of Medical Sciences, Isfahan, Iran; ^4^Department of Clinical Pathology, School of Medicine, Isfahan University of Medical Sciences, Isfahan, Iran

## Abstract

**Background:**

Gamma-aminobutyric acid (GABA) and magnesium sulfate (MgSO_4_) play a crucial role in glycemic control. Therefore, we studied the effect of combination therapy with GABA and MgSO_4_ to improve insulin sensitivity in diabetes induced by streptozotocin as well as high-fat diet in a diabetic rat model. *Design and Methods*. Forty randomly selected rats were assigned to four groups: nondiabetic control group was fed the normal diet, insulin-resistant diabetic rat model was induced by streptozotocin and high-fat diet, GABA + MgSO_4_ group received GABA and MgSO_4_, and insulin group was treated with insulin. Body weight, abdominal fat, blood glucose, serum insulin, and glucagon concentration were measured. The glucose clamp technique, glucose tolerance test, and insulin tolerance test were performed to study insulin sensitivity. Also, the expressions of glucose 6 phosphatase, glucagon receptor, and phosphoenolpyruvate carboxykinase genes in liver were assessed for the gluconeogenesis pathway. Protein translocation and glucose transporter 4 (*Glut4*) genes expression in muscle were also assessed.

**Results:**

Combination of GABA + MgSO_4_ or insulin therapy enhanced insulin level, glycemic control, glucose and insulin tolerance test, some enzymes expression in the gluconeogenesis pathway, body fat, body weight, and glucagon receptor in diabetic rats. Moreover, an increase was observed in protein and gene expression of *Glut4*. Insulin sensitivity in combination therapy was more than the insulin group.

**Conclusions:**

GABA and MgSO_4_ enhanced insulin sensitivity via increasing *Glut4* and reducing the gluconeogenesis enzyme and glucagon receptor gene expressions.

## 1. Introduction

World Health Organization (WHO) announced that, by 2030, diabetes is going to be the seventh major cause of mortality [1]. Hyperglycemia as one of diabetes pathogenesis is caused by several factors such as impaired insulin secretion, or PI3/AKT signaling [2], which leads to the impairment of the insulin-dependent glucose transporter 4 (Glut4) in muscles and hence the utilization of glucose decreases which is called peripheral insulin resistance [3, 4]. Another important pathophysiology of diabetes is increasing glucagon secretion despite hyperglycemia [5]. In addition to lipolysis [6], the main target of glucagon is *G* protein-coupled receptors on the plasma membrane of hepatocytes [5] which finally upregulates phosphoenolpyruvate carboxykinase (PEPCK) and glucose 6 phosphatase (G6Pase), the key enzymes in gluconeogenesis, leading to an increase in hepatic glucose output; thus, an increase in gluconeogenesis is linked to the overexpression of PEPCK and G6Pase [5].

Many antidiabetic drugs with different target receptors are being used in the clinic right now to decrease blood glucose [5]. Because type 2 diabetes mellitus (T2DM) is a complicated disease, patients are usually treated with a mixture of antidiabetic drugs. For several reasons, different drugs with complementary mechanisms of action are often needed to be used. Because the disease is so progressive, and glycemic control over time is impaired, so monotherapy fails to keep glycemic under control in the long run [7]. Therefore, nowadays, multidrug therapies are reinvestigated; [8] it is believed that combination therapies with diverse mechanisms may also be a first-line choice [9]. Antidiabetic drugs mechanisms include reducing insulin resistance, diminishing hepatic glucose production, enhancing endogenous insulin secretion, and limiting glucose reabsorption [5].

Our earlier study and other investigations [10–14] showed that dietary magnesium (Mg) has a strong effect on T2DM and insulin resistance. These improvements have been achieved by some mechanisms like increasing *Glut4* gene expression independent of insulin secretion [15]. Therefore, Mg could improve glucose metabolism [16]. But, in our previous study, Mg could not improve insulin secretion [11, 15].

Also, gamma-aminobutyric acid (GABA), produced from glutamate in islet beta cells, is coreleased with insulin. GABA has an influence on both beta and alpha cells with different actions. In beta cells, it provokes the depolarization of membrane, thereby augmenting calcium entering the cells and finally resulting in insulin secretion. However, in alpha cells, GABA results in membrane hyperpolarization reducing glucagon secretion [17]. Keeping the perfect GABA levels may be essential in glucose metabolism control [18]. Studies have shown protective action of GABA in type 1 diabetes mellitus (T1DM) and T2DM [19–22] with different mechanisms including regenerative and protective effects on beta cells, suppressing insulitis and systemic inflammatory cytokine production, and decline regulatory T-cells. GABA-treated animals showed higher plasma insulin, falling glucagon level, normalized glycemic condition, and enhanced metabolic state [23].

In our previous study, GABA or MgSO_4_ could improve resistance to insulin. Hence, the present study aims to compare the efﬁcacy of combination therapy through GABA and Mg with complementary mechanisms versus maximum insulin dose in a chronic insulin-resistant high-fat type 2 diabetic rats model, which is the most similar to human T2DM [24]. We also evaluated some key gene expression in glucose metabolism and insulin resistance.

## 2. Subjects and Methods

### 2.1. Animals

All experiments were performed in compliance with NIH guidelines for the animals' care and control. They were also approved by the ethics committee of Isfahan University of Medical Sciences in Iran (ethic code IR.MUI.REC.1394.3.766). Forty age-matched male Wistar rats were housed in cages (five rats per cage) in a room with controlled temperature, 12-hour light/12-hour dark cycles, and free access to food and water.

### 2.2. Experimental Protocol

Young rats, after weaning (30 day), were fed with a regular pellet diet, or high-fat diet (HFD). HFD consists of regular pellet diet powdered = 365 g/kg, lard = 310 g/kg, casein = 250 g/kg, cholesterol = 10 g/kg, vitamin, and mineral mix = 60 g/kg, DL-methionine = 3 g/kg, yeast power = 1 g/kg, and sodium chloride = 1 g/kg [11, 25, 26].

After one month, rats with HFD were injected with 35 mg/kg of streptozotocin (STZ) intraperitoneally (I.P) (Sigma Aldrich, Hamburg, Germany) and rats in the control group received normal saline. Three days later, we sued T2DM models for groups of animals that had fed blood glucose level (at 9 AM) greater than 250 mg/dl for three successive days and they were fed with the same individual diets up to 12 weeks (HFD: 4920 vs. 3100 Kcal/kg).

Animals were randomly assigned to four groups (*n* = 10): the chronic diabetic group (D), the nondiabetic control group (NDC) fed with a regular diet, diabetic animals receiving regular and NPH insulin (2.5 U/Kg two times per day) (Ins-D), and animals with diabetes receiving GABA [20] treatment (1.5 g/kg, I.P) + MgSO_4_ [27] (10 g/l add in drinking water) (GABA + Mg-D). Throughout the course of treatment, animals were regularly weighed and their level of blood glucose was measured using an EasyGluco glucometer every week.

### 2.3. Metabolic Measurements

The intraperitoneal glucose tolerance test (GTT) was conducted at the eleventh week of diabetes induction. As previously published [11, 20], after 15 h of fasting, basal blood samples were taken and 1.5 g/kg body weight *D* (+)-glucose subsequently were administered via I.P injection. Blood was taken from a cut at the tip of the tail at 0, 10, 20, 30, 60, and 120 min.

For the insulin tolerance test (ITT), animals received 2.5 U/kg body weight insulin regular (Exir pharmaceuticals, Lorestan, Iran) via I.P injection. Blood was drawn from the tail vein in different time intervals after following insulin administration (0, 20, 30, 40, 60, 90, and 120 min after) and blood glucose levels were determined. It was carried out 5 days before the end of the study [11, 20].

### 2.4. Insulin Sensitivity

At the end of 12-week experiment, rats were fasted overnight. They were then anesthetized with xylazine (8 mg/kg I.P), ketamine (100 mg/kg I.P), and 30 mg/kg injection of pentobarbital sodium every 30 min.

The cannulation of trachea was conducted for safe respiration. The cannulation of left carotid artery (to withdraw blood) and the right jugular vein (for glucose infusion and insulin) was carried out by polyethylene tubing (PE 9658, Microtube Extrusions, North Rocks NSW, Australia). Following the injection of a bolus dose of insulin (100 mU/kg) (Exir pharmaceuticals, Lorestan, Iran), insulin was infused continually (20 mU/kg/min) by an infusion pump (KB Scientific, Iran) to maintain blood glucose at the basal level throughout the 120 min of the experiment. We used a glucometer to take blood sample for testing glucose concentration every 5–10 min followed by the infusion of glucose solution (25%) in clamp euglycemia at 100 mg/dl ± 5 level.

Finally, the insulin-stimulated glucose uptake was estimated (by the rate of glucose infusion during the ﬁnal 30 minutes of the clamp). The whole-body insulin sensitivity was determined by the glucose infusion rate (GIR: mg kg-1 min-1) [11, 20, 28].

After completing the clamp procedure, blood samples were accumulated using the cardiac puncture and transferred to a tube after separating serum via centrifugation at 1968 g for 10 min. The gathered serum was saved at −80°C for biochemical evaluation.

### 2.5. Measurement of Blood Metabolic Parameters

ELISA kit particularly designed for rats (Mercodia AB, Uppsala, Sweden Elisa kit, ZellBio GmbH, Germany, resp.) was used for assessing fasting serum insulin and glucagon concentrations that were read by an automatic plate reader (Stat fax-2100, Beiken Company). Serum Mg levels were estimated by the commercial kit (Greiner kits with the Arsenio method, resp.) according to instructions provided by the manufacturer and Hitachi 717 auto analyzer (Roche Diagnostics, Basel, Switzerland).

### 2.6. Quantitative Real-Time PCR

At the end of 12 weeks of treatment, after 120-minutes, hyperinsulinemic-euglycemic clamp, the left gastrocnemius muscle (for Glut4), and the median lobe of the liver for glucagon receptor and G6Pase and PEPCK gene expression were detached. Afterward, 50–100 mg of tissue was placed in RNase later (Qiagen, Hilden, Germany) and saved at −80°C prior to the isolation of RNA.

The total RNA was isolated by the Iraizol kit (RNA Biotech.IRAN) according to the instructions provided by the manufacturer. To produce complementary DNAs (cDNA), 1 *µ*g of total RNA was utilized by RB MMLV (RNA Biotech.IRAN Kit). The SYBR green was also used for quantitative real-time PCR using the Step-One Real-Time PCR machine (Applied Biosystems). In the next step, we mixed 1 *µ*l of total cDNA with treated water, 10 *µ*l SYBR Green, and 10 pmol/ml of sense and antisense primers for the evaluated gene. The primers sequences included Glucagon receptor (NM_172092) sense primer: GGATCCTGCGTATCCCTGTA and antisense primer: GCTCATCAGTCACAAAGGCA [29]; G6Pase(NM_017006) sense primer: GCGACCGTCCCCTTTGCATCTGTC and antisense primer: CCACCAAACACTCCCCCTCCTCC [30]; Glut4(NM_012751) sense primer: GTGTGGTCAATACCGTCTTCACG and antisense primer: CCATTTTGCCCTCAGTCATTC (designed with NCBI's primer BLAST); PECPK (NM_198780) sense primer: GCCTCCTCAGCTGCATAATGGTCT and antisense primer: GAATGCTTTCTCGAAGTCCTCTTCTG (designed with NCBI's Primer BLAST); beta-actin sense primer: AGGCCCCTCTGAACCCTAAG and antisense primer: CCAGAGGCATACAGGGACAA [31]. Primers were analyzed with NCBI primer BLAST and OLIGO 7 software to ensure their quality and suitability like primer melting temperatures and G/C content (all primers were made by Pishgam Biotech Company, Tehran, Iran).

As an internal reference gene, the mean expression of beta-actin was utilized and the mRNA expression of target genes was estimated relative to beta-actin. The relative expression values were measured by the comparative CT method and the ∆Ct value was used to plot the relative gene level estimated by the expression 2^−∆∆Ct^.

### 2.7. Glut4 Protein Expression by Immunohistochemistry

After completing the euglycemic hyperinsulinemic clamp, the rapid removal of the right gastrocnemius muscle was performed followed by fixation in 10% solution of formaldehyde, and building of paraffin blocks. The sections fixed in formalin and embedded in paraffin were processed through a graded alcohol series and xylene and then incubated with 25% H_2_O_2_. After that, the incubation of slides was carried out using anti-Glut4 antibody of rats (a mouse monoclonal antibody) at 500 *μ*g/ml (Santa Cruz Biotechnology, Santa Cruz, CA, Glut4 [(IF8): sc-53566] and washed with PBS; then, the slides were treated with secondary antibody and so nourished with streptavidin–HRP (Jackson Labs, West Grove, PA). Then, the processing of slides was conducted with AEC reagent (Zymed Laboratories, San Francisco, CA) followed by the counterstaining of hematoxylin (Sigma Co., St. Louis, MO). Finally, cells were investigated and the images were captured under the bright field [20].

### 2.8. Measurement of the Urine Output, Food Intake, and Water Intake

The rats were moved to a clean empty metabolic cage once at a time by day (Aratebfan Co., Tehran, Iran). The food intake, water drinking, and urine volume were measured.

### 2.9. Measurement of Abdominal Fat Mass

All intra-abdominal fat including retroperitoneal and periepididymal fat was excised and weighed upon sacrifice. Then, wet weights of organ were defined as visceral fat mass.

### 2.10. Statistics

Data were expressed as mean ± SEM. For statistical comparison among groups, we used Tukey post-hoc test along with one-way and two-way analysis of variance (ANOVA). The target gene's expression pattern was studied using the Spearman and Mann-Whitney *U*-tests. *P*-values<0.05 were considered significant and data analysis was conducted using SPSS (22) software.

## 3. Results

### 3.1. Effect of GABA + MgSO4 on Plasma Glucose, ITT, GTT, and GIR

**Figure 1:**
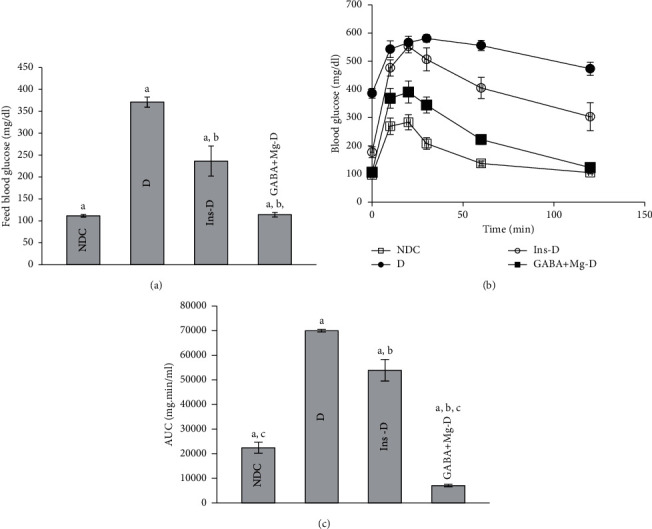
Comparison of feed blood glucose. (a) intraperitoneal glucose tolerance test (GTT) and (b) the area under the curve (AUC) of blood glucose 3 months after treatment. (c) In the control group (NDC-without diabetes) receiving a normal diet, the group with chronic diabetic were fed a high-fat diet along with 35 mg/Kg STZ (d), diabetic animals receiving insulin treatment (2.5 U/Kg two times per day) (Ins-D), and animals with diabetes receiving GABA treatment (1.5gr/kg)+MgSO_4_ (10 g/l add in drinking water) (GABA + Mg-D) (each group has 10 rats. Data are presented as mean ± SEM). A significant difference between D and other groups (*P* < 0.0001). b Significant difference between GABA + Mg-D and Ins-D group (*P* < 0.001). c Significant difference between NDC group and GABA + Mg-D (*P* < 0.01).

Feeding blood glucose was evaluated at the conclusion of treatment in all groups (Figure 1(a)). The GABA + Mg or insulin injection for 12 weeks reduced the concentration of plasma glucose in the GABA + Mg-D and Ins-D groups compared with *D* group (113.8 ± 2.41 mg dl^−1^; 236.38 ± 34.38 mg dl^−1^; 371.11 ± 11.65 mg dl^−1^, in the respective order, in the week 12, *p* < 0.0001). There was a substantial difference between GABA + Mg-D and Ins-D groups (*p* < 0.001). GABA + Mg decreased plasma glucose level similar to the control group and more than insulin group (NDC; 111 ± 2.9 mg dl^−1^). Data regarding changes in weekly blood glucose were provided as Figure 1 in supplementary materials.

GTT was impaired in diabetic group in comparison to diabetic rats treated with GABA and Mg or insulin (D: 69992.86 AUC (area under curve) ± 551.73 vs. GABA + Mg-D: 7066.25 AUC ± 527.56, Ins-D: 53915.71 AUC ± 4356.783, NDC: 22406.36 AUC ± 2273.45, *P* < 0.0001). In GABA + Mg-D group, glucose AUC was lower compared to insulin-treated group (*P* < 0.001, Figures 1(b) and 1(c)).

**Figure 2:**
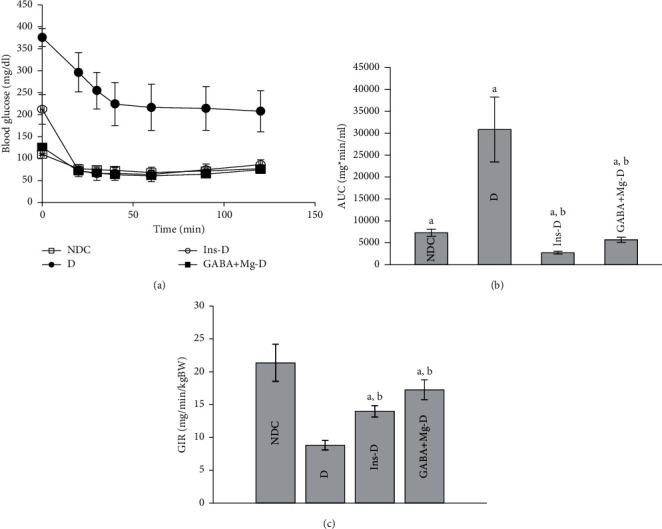
Comparison of insulin tolerance test (a) and the area under the glycemic curve (AUC) (b) and GIR (c) 3 months after treatment in the nondiabetic control group (NDC) who received a normal diet, the group with chronic diabetic fed high-fat diet and 35 mg/Kg STZ (d), diabetic animals treated with insulin (2.5  U/Kg two times per day) (Ins-D), and animals with diabetes receiving GABA treatment GABA (1.5gr/kg) + MgSO_4_ (10 g/l add in drinking water) (GABA + Mg-D) (10 rats in each group. Data are expressed as mean ± SEM). (a) Significant difference between D and other groups (*P* < 0.0001). (b) Significant difference between GABA + Mg-D and Ins-D group (*P* < 0.001). (c) Significant difference between NDC group and GABA + Mg-D (*P* < 0.01).

After 3-month treatment, ITT was carried out in all groups. GABA + Mg treated diabetic rats showed improved levels of insulin sensitivity and blood glucose in comparison with *D* animals (*P* < 0.001, Figures 2(a) and 2(b)). However, Ins-D had the maximum improvement (glycemic response was expressed as AUC : GABA + Mg-D: 5689.37 ± 552.2, vs. D: 30849.29 ± 7408.66; Ins-D: 2730 ± 318.15; NDC: 7291.2 ± 803.05 *P* < 0.01).

After the study, the hyperinsulinemic-euglycemic clamp test was carried out in all animals to evaluate insulin sensitivity in whole body. The clamping of blood glucose was conducted at 100 mg dl^−1^ ± 5. The glucose infusion rate (GIR) was significantly raised by the GABA + Mg treatment, which is essential to preserve euglycemia at the time of insulin infusion in comparison with diabetic rats (D: 8.81 ± 0.73 mg kg^−1^ min^−1^; GABA + Mg-D: 17.25 ± 1.5 mg kg^−1^ min^−1^, *P* < 0.0001). GIR was higher in insulin-treated rats than in the diabetic group (Ins-D: 13.97 ± 0.86 mg kg^−1^ min^−1^). Nevertheless, most of GIR was seen in GABA + Mg-D rats compared to the insulin group (*P* < 0.001, Figure 2(c)).

### 3.2. Effect of GABA + MgSO_4_ on Changes in Blood Magnesium, Insulin, Glucagon Levels, and Glucagon Receptor mRNA Gene Expression in Liver

**Figure 3:**
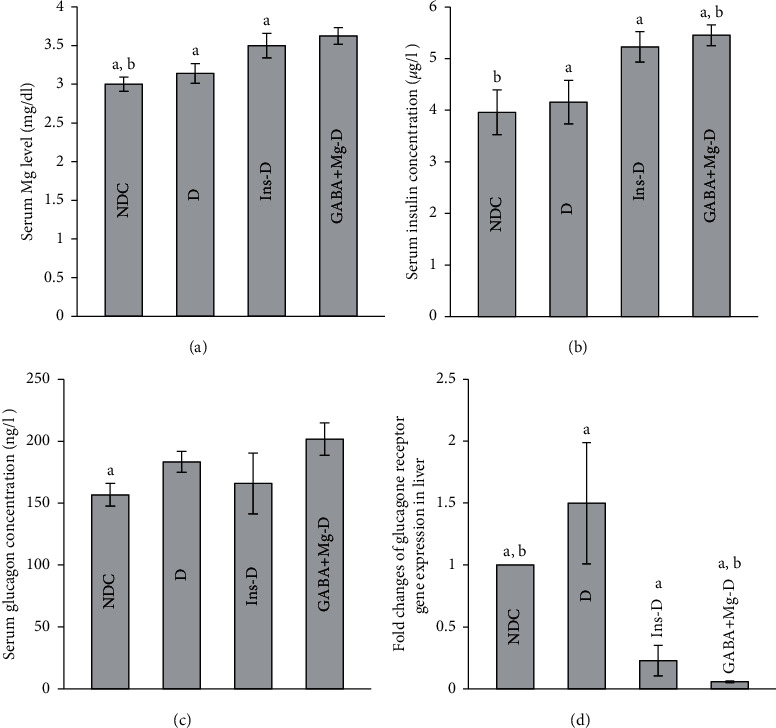
Comparison of serum magnesium level. (a) Glucagon, (b) insulin concentrations, (c) and gene expression of glucagon receptor in liver. (d) Control group without diabetes was fed with a normal diet, the chronic diabetic group received high-fat diet and 35 mg/Kg STZ (D), diabetic animals were treated with insulin (2.5 U/Kg two times per day) (Ins-D), and diabetic animals treated with GABA (1.5gr/kg) +MgSO_4_ (10 g/l add in drinking water) (GABA + Mg-D) (10 rats in each group. data are expressed as mean ± SEM). (a) Significant difference between D and other groups (*P* < 0.001). (b) Significant difference between NDC group and GABA + Mg-D (*P* < 0.01).

As shown in Figure 3(a), Mg levels in different treatment groups were more than diabetic rats (GABA + Mg-D: 3.62 ± 0.1 mg dl^−1^, Ins-D: 3.5 ± 0.15 mg dl^−1^, D: 3.14 ± 0.12 mg dl^−1^, *P* < 0.001); and control rats has least concentration (NDC: 3 ± 0.09 mg dl^−1^, *P* < 0.01). The glucagon level and fasting serum insulin were evaluated in all groups after 12-week diabetes treatment and induction. Serum glucagon level was considerably higher in *D* animals than in the NDC group (*P* < 0.001, D: 183.3 ± 8.45 ng l^−1^; NDC: 156.7 ± 9.2 ng l^−1^). GABA + Mg and insulin could not suppress glucagon level (*P* < 0.001) (GABA + Mg-D: 201.68 ± 13.18 ng l^−1^; Ins-D: 165.8 ± 24.5 ng l^−1^, Figure 3(c)). Following the induction of chronic diabetes, the concentration of blood insulin in *D* rats dropped significantly (*P* < 0.001, Figure 3(b)). In all treated rats, insulin level spiked, but the insulin level in the GABA + Mg group was elevated more than the control group (*P* < 0.01, NDC: 3.96 ± 0.43 *µ*g l^−1^; GABA + Mg-D: 5.45 ± 0.2 *µ*g l^−1^; Ins-D: 5.23 ± 0.29 *µ*g l^−1^; D: 4.15 ± 0.42 *µ*g l^−1^). Glucagon receptor mRNA gene expression in liver declined considerably in all treated animals in compared to the diabetic rats (Figure 3(d), *P* < 0.001) but no difference was observed between treatment groups.

### 3.3. Effect of GABA + MgSO_4_ on Body Weight Change and Fat Distribution

Body weight was determined on a weekly basis. Diabetic rats' weight at week 12 almost did not change, but it significantly increased in all treated animals (*P* ≤ 0.001) (NDC: 351.42 ± 11.67 g, D: 267 ± 12.97 g; vs. Ins-D: 320.5 ± 12.91 g; GABA + Mg-D: 323 ± 25.48 g; *P* < 0.001, Figure 2(a) in supplementary materials). No difference was observed between treatment groups (*P* > 0.5). In diabetic group, abdominal fat mass reduced (NDC: 0.023 ± 0.002 g kg^−1^, D: 0.0092 ± 0.001 g kg^−1^), but fat mass was considerably higher in the treatment groups than in diabetic rats. Between two treatment groups compared to control rats, the insulin group had the highest fat mass (*P* < 0.01, GABA + Mg-D: 0.016 ± 0.001 g kg^−1^; Ins-D: 0.026 ± 0.001 g kg^−1^, Figure 2(b) in supplementary materials).

### 3.4. Effect of GABA + MgSO_4_ on Food and Fluid Intake and Urine Flow Rate

**Figure 4:**
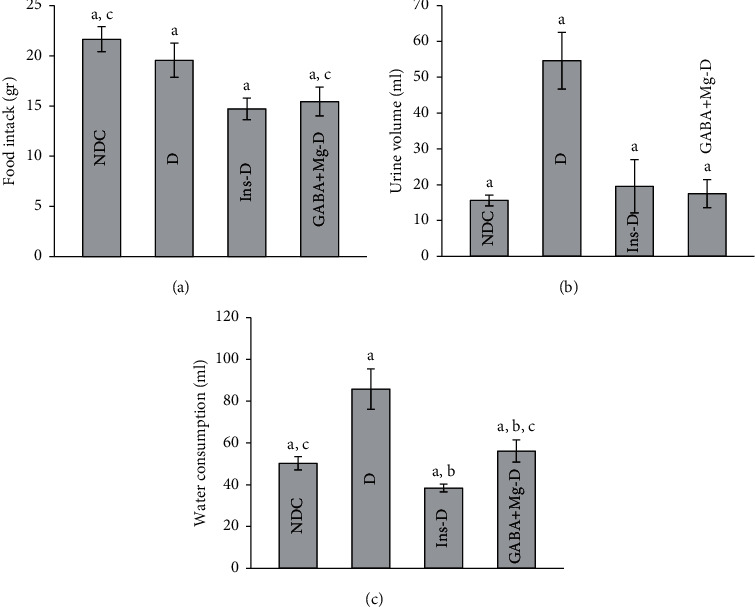
Comparison of food intake (a), urine volume (b), and water consumption. (c) In the nondiabetic control group (NDC) fed with a normal diet, the chronic diabetic group received high-fat diet and 35 mg/Kg STZ (D), diabetic animals treated with insulin (2.5 U/Kg two times per day) (Ins-D), and diabetic animals treated with GABA (1.5gr/kg) + MgSO_4_ (10 g/l add in drinking water) (GABA + Mg-D) (10 rats in each group; data are expressed as mean ± SEM). (a) Significant difference between D and other groups (*P* < 0.001). (b) Significant difference between GABA + Mg-D and Ins-D group (*P* < 0.01). (c) Significant difference between NDC group and GABA + Mg-D (*P* < 0.01).

In the untreated diabetic group, the urine flow rate and food and fluid intake were greater than other treated groups. The consumption of foods was also higher in control rats than that in treated animals (Figure 4(a). The insulin injection or GABA + Mg enhanced metabolic cage conditions in diabetic rats (Figure 4(a)–4(c)*P* < 0.001). Except for water consumption, no significant differences were observed in other metabolic findings obtained from cage between treatment groups.

### 3.5. Effect of GABA + MgSO_4_ on PEPCK and G6Pase mRNA Gene Expressions

**Figure 5:**
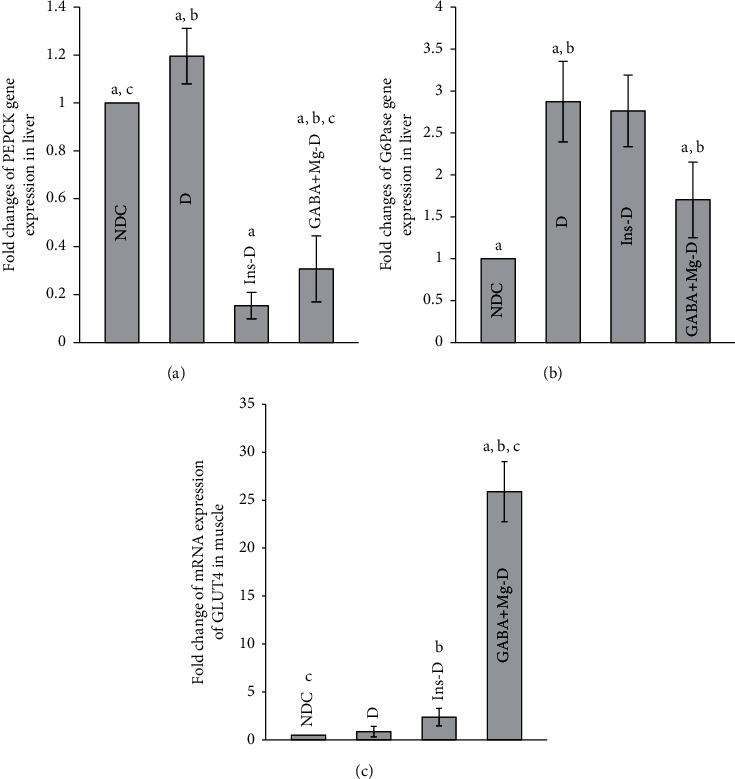
Comparison of fold change in PEPKC mRNA expression. (a) G6pase, (b) mRNA expression of GLUT4, (c) the nondiabetic control group (NDC) was fed with a normal diet, the chronic diabetic group received high-fat diet and 35 mg/Kg STZ (D), diabetic animals treated with insulin (2.5 U/Kg two times per day) (Ins-D), and diabetic animals treated with GABA (1.5gr/kg) + MgSO_4_ (10 g/l add in drinking water) (GABA + Mg-D) (10 rats in each group. Data are expressed as mean ± SEM). (a) Significant difference between D and other groups (*P* < 0.01). (b) Significant difference between GABA + Mg-D and Ins-D group (*P* < 0.01). (c) Significant difference between NDC group and GABA + Mg-D (*P* < 0.01).

G6Pase and PEPCK mRNA gene expressions are shown in Figure 5. PEPCK mRNA expression declined in all animals in the treatment group in compared to diabetic rats (*P* < 0.001). No differences were observed among treated groups (Figure 5(a)). G6Pase gene expression reduced considerably in GABA + Mg-D group in compared to diabetic rats (*P* < 0.01), but *D* and the insulin-treated groups were not significantly different (Figure 5(b)).

### 3.6. Effect of GABA + MgSO_4_ on Variations in GLUT4 mRNA Gene Expression and Its Protein Level in Muscle

**Figure 6:**
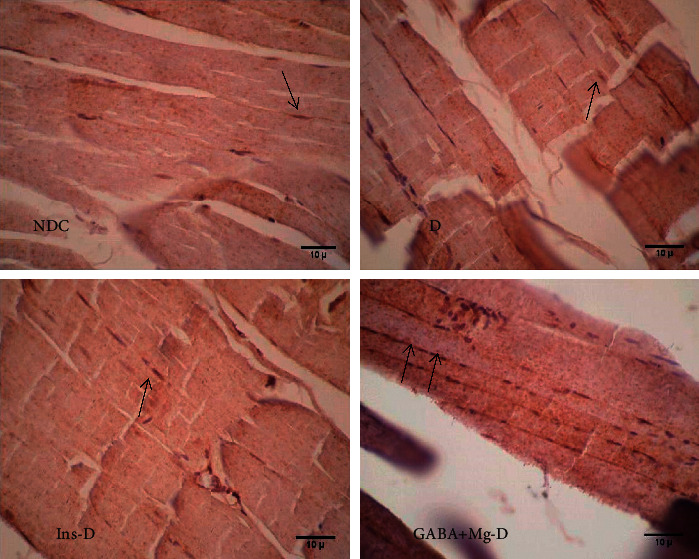
Comparison of immunohistochemistry for GLUT4 in the nondiabetic control group (NDC) fed with a normal diet, the chronic diabetic group received high-fat diet and 35 mg/Kg STZ (D), diabetic animals treated with insulin (2.5 U/Kg two times per day) (Ins-D), and diabetic animals treated with GABA (1.5gr/kg) + MgSO_4_ (10 g/l add in drinking water) (GABA + Mg-D) (10 rats in each group. Data are expressed as mean ± SEM) (X400, black arrows indicate the translocation of GLUT4 to the cell membrane).

A 30-fold surge in GLUT4 mRNA was observed in rats treated with GABA and Mg, along with a 2.8-fold in the insulin group (*P* < 0.001), in comparison with the *D* group. The Glut4 mRNA expression was significantly upregulated by GABA + Mg treatment in comparison with the insulin group (*P* < 0.01, Figure 5(c)).  Figure 6(a) displays that the protein level of Glut4 in the cell membrane spiked in GABA + Mg-D group compared to the other groups.

## 4. Discussion

We previously studied the hypoglycemic effect of Mg on the T1DM rats' model, T2DM rats and patients [10, 11, 32, 33]. The strongest recorded effect of Mg was through increased Glut4 in muscles [11, 15]. In addition, we studied the GABA effect on T1DM mouse models and T2DM rats [19, 20]. GABA could improve pancreatic cell protection and insulin secretion [19–35]. Our studies showed that GABA and magnesium not only improved glucose metabolism in diabetic rats but also increased insulin sensitivity in their offspring [33, 34].

Therefore, we hypothesized that administering Mg through acting on muscle combined with GABA that could improve pancreatic function may synergistically inhibit insulin resistance and promote restoration of normoglycaemia in a chronic T2DM rat model. Concurrent use of different antidiabetic drugs to achieve glycemic control is one of the treatment strategies for T2DM [36].

The results showed that, following diabetes induction, a 12-week high-fat diet induced glucose intolerance and insulin resistance in rats compared to control ones. Mg + GABA or insulin administration in comparison with the untreated group enhanced insulin resistance, blood glucose, GTT, weight, level of plasma insulin, and body fat percentage in diabetic rats.

The improvements in insulin sensitivity, blood glucose, and GTT following chronic Mg + GABA treatment might be caused by direct insulin elevation or indirect suppression of hepatic glucose production. In addition, we did not study pancreatic histology and this is our study limitation; however, according to our previous investigation and other studies [19, 37], blood glucose was reduced by GABA therapy through insulin secretion improvement with beta cells protection. So elevated insulin secretion in this study with GABA treatment may be followed by GABA inserted beta cell protection and decreased beta cell apoptosis. Despite the fact that insulin concentration between the GABA + Mg group and insulin-treated rats was not different, finally combination-treated rats had better insulin sensitivity and less peripheral resistance with high GIR and improving the ITT test. So, this may not be related to insulin secretion alone. Some other factors probably contribute to improving insulin sensitivity. Since diabetes is a bihormonal disease that not only impaired insulin action but also elevated glucagon resulting in diabetic complication [23], the study reported that dysregulation of glucagon secretion has also been involved in the pathophysiology of T2DM [38]. According to Lee et al. [39], in diabetic mice if glucagon is suppressed, blood glucose normalizes regardless of complete insulin absent. In Feng et al.'s study [17], treatment with insulin neither prevented the proliferation of alpha cell nor induced protection against beta cell injury. They reported that insulin therapy alone without GABA via activation of mTOR could increase *α* cell mass so increasing glucagon secretion. It is similar to our study that insulin therapy could not decrease serum glucagon levels in diabetic rats. Moreover, in our previous study we reported that glucagon concentrations were not suppressed in GABA-treated rats [20] maybe because of short time of study, but glucagon receptor was surprisingly decreased. This decrease may be secondary to insulin secretion in GABA + Mg treated rats or other factors that need more investigations.

PEPCK and G6Pase are two crucial hepatic enzymes that in response to glucagon dramatically increase gluconeogenesis and blood glucose. Glucagon is the major parameter in the development of diabetes [5, 6]. In mice without hepatic glucagon receptor and with insulin deficiency, hepatic levels of *PEPCK* mRNA were profoundly reduced but after the insertion of glucagon receptor, blood glucose elevated again because of increased levels of *PEPCK* mRNA [39]. Thus, it seems that glucagon is essential for hyperglycemia and in the absence of glucagon action, the liver fails to produce excess glucose [5, 40]. Although, in our treated groups, serum glucagon level did not decrease, the combination therapy decreased glucagon receptor mRNA expression. Therefore, in spite of glucagon elevation, it could not increase blood glucose. This might be linked to insulin secretion because in insulin-treated rats, the glucagon receptor also decreased, but it should be investigated further. However, glucagon in two treated insulin and GABA + Mg groups was not reduced, and because of glucagon receptor downregulation, *PEPCK* and *G6Pase* mRNA decrease, and blood glucose tends to normalize.

The evidence from this study suggests that, after Mg + GABA administration, ITT and GTT improved significantly. However, the ITT in insulin group was better than Mg + GABA group, but GIR which is more sensitive than ITT [41] was considerably more in Mg + GABA group which may be due to 10-fold elevating muscle *Glut4* mRNA in this group compared to insulin group. Further research with euglycemic hyperglycaemic clamps and administration of glucose isotope is warranted to clarify the exact role of liver and muscle in improving insulin resistance.

Recent research suggests that GABA can boost beta-cell replication and survival [39]. Thus, in this study GABA's impact on insulin resistance is likely secondary to the metabolic control enhancement through pancreas action. Our findings showed that Mg + GABA treatment increased insulin secretion which may be due to improvement in function of beta cells. Although in our study increased insulin did not inhibit glucagon secretion, lack of the enough time may be the reason. Yet, this combination therapy decreased glucagon receptor mRNA expression. Therefore, glucagon could not increase blood glucose. Diabetes is a complex and bihormonal disease. The ratio of insulin to glucagon determines normal blood glucose level. The study has shown that, in diabetics, decrease in insulin is not as harmful as an increase in glucagon, as insulin deficient animals that were simultaneously knocked out of the glucagon receptor were normoglycemic [39].

Furthermore, this combination therapy decreased *PEPCK* and *G6Pase* gene expressions (more than insulin groups). We observed the enhancement of hepatic insulin sensitivity is also likely since coadministration of Mg + GABA decreased key gluconeogenic enzyme gene expression.

## 5. Conclusions

Our observations suggest that combined use of GABA and magnesium in T2DM treatment is feasible, which leads to the improvement of insulin resistance in high-fat STZ-induced T2DM as well as additive therapeutic effects on metabolic key enzymes gene expression. GABA and Mg can also be orally administered [10, 42, 43], and this is an important clinical advantage. Further preclinical and clinical trials are suggested to test the efficacy of combined use of GABA and Mg in humans.

## Data Availability

The data used to support the findings of this study are included within the article.

## Supplementary Materials

Supplementary Materials Supplementary materials include two files. The first file contains Supplementary Figure 1: comparison of feed blood glucose. The second file contains Supplementary Figure 2: comparison of body weight (a), and abdominal fat (b).
