# Supplementary figures and images for: Combination Therapy with GABA and MgSO4 Improves Insulin Sensitivity in Type 2 Diabetic Rat
Source: Int J Endocrinol. 2022 Feb 15;2022:2144615. doi: 10.1155/2022/2144615 (PMC8863457; doi:10.1155/2022/2144615)

## Supplementary

FIGURE 1

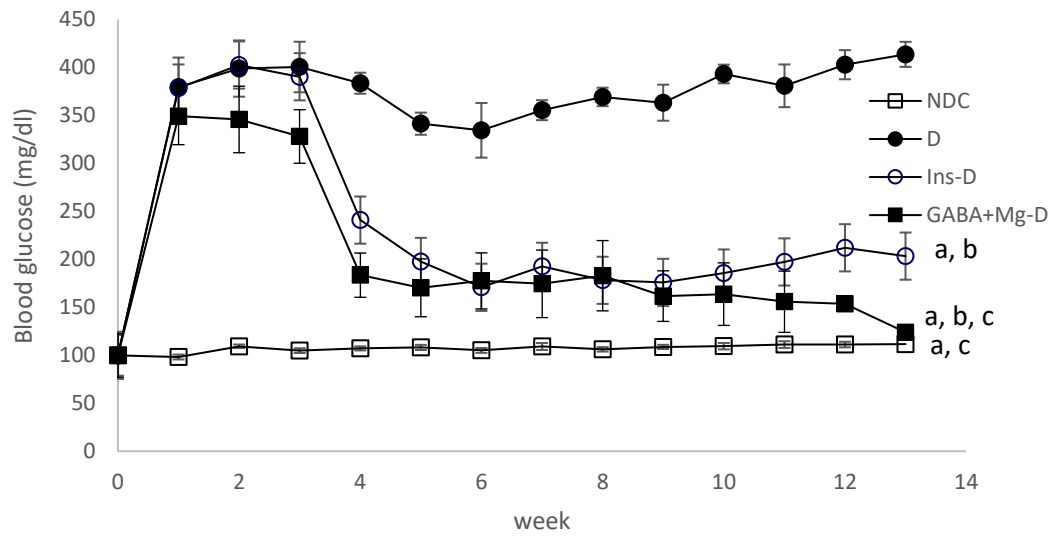

FIGURE 2

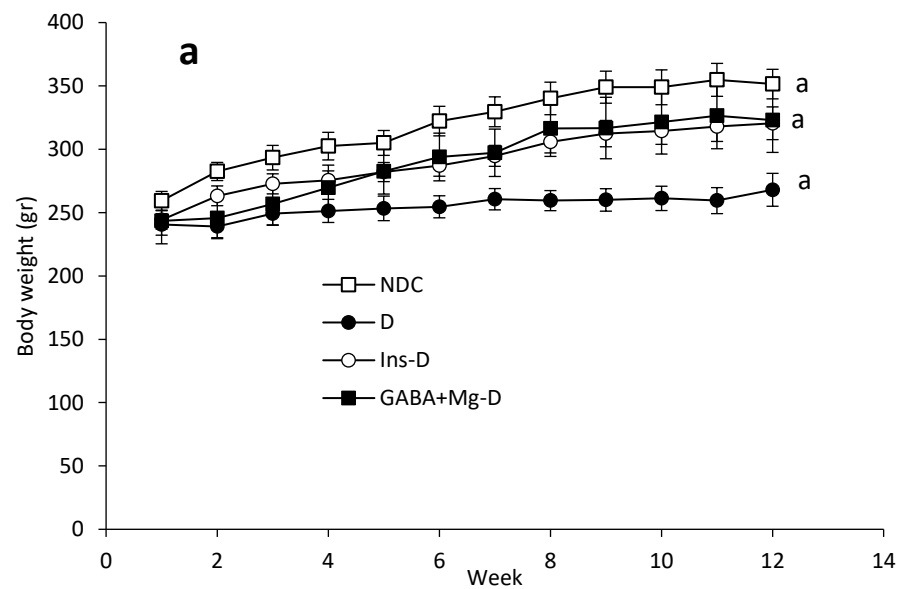

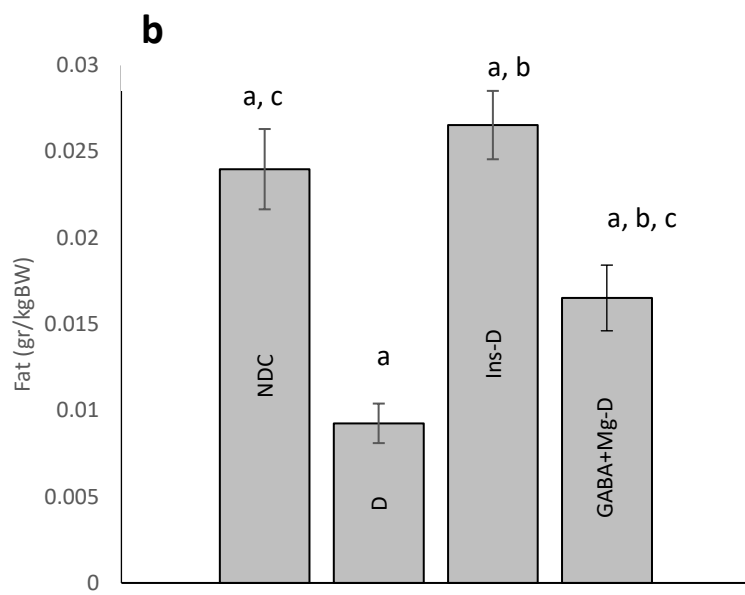

Supplement: Supplementary Materials — include two files. The first file contains Supplementary Figure 1: comparison of feed blood glucose. The second file contains Supplementary Figure 2: comparison of body weight (a), and abdominal fat (b). [file 2144615.f1.pdf]
